# TIPRL, a Novel Tumor Suppressor, Suppresses Cell Migration, and Invasion Through Regulating AMPK/mTOR Signaling Pathway in Gastric Cancer

**DOI:** 10.3389/fonc.2020.01062

**Published:** 2020-07-03

**Authors:** Meng Luan, Shan-Shan Shi, Duan-Bo Shi, Hai-Ting Liu, Ran-Ran Ma, Xiao-Qun Xu, Yu-Jing Sun, Peng Gao

**Affiliations:** ^1^Key Laboratory for Experimental Teratology of Ministry of Education, Department of Pathology, School of Basic Medical Sciences, Cheeloo College of Medicine, Shandong University, Jinan, China; ^2^Institute of Basic Medicine, Shandong Provincial Hospital Affiliated to Shandong First Medical University, Jinan, China; ^3^Key Laboratory of Cardiovascular Proteomics of Shandong Province, Department of Geriatrics, Qilu Hospital of Shandong University, Jinan, China; ^4^Department of Pathology, Qilu Hospital, Shandong University, Jinan, China

**Keywords:** TIPRL, gastric cancer, invasion, metastasis, AMPK/mTOR signaling

## Abstract

Invasion and metastasis of gastric cancer after curative resection remain the most common lethal outcomes. However, our current understanding of the molecular mechanism underlying gastric cancer metastasis is far from complete. Herein, we identified TOR signaling pathway regulator (TIPRL) as a novel metastasis suppressor in gastric cancer through genome-wide gene expression profiling analysis using mRNA microarray. Decreased TIPRL expression was detected in clinical gastric cancer specimens, and low TIPRL expression was correlated with more-advanced TNM stage, distant metastasis, and poor clinical outcome. Moreover, TIPRL was identified as a direct target of miR-216a-5p and miR-383-5p. Functional study revealed that re-expression of TIPRL in gastric cancer cell lines suppressed their migratory and invasive capacities, whereas inverse effects were observed in TIPRL-deficient models. Mechanistically, TIPRL downstream effectors and signaling pathways were investigated using mRNA microarray. Gene expression profiling revealed that TIPRL could not modulate the downstream genes at transcriptional levels, thereby implying that the regulation might occur at the post-transcriptional levels. We further demonstrated that TIPRL induced phosphorylation/activation of AMPK, which in turn attenuated phosphorylation of mTOR, p70S6K, and 4E-BP1, thereby leading to inactivation of mTOR signaling and subsequent suppression of cell migration/invasion in gastric cancer. Taken together, TIPRL acts as a novel metastasis suppressor in gastric cancer, at least in part, through regulating AMPK/mTOR signaling, likely representing a promising target for new therapies in gastric cancer.

## Introduction

Gastric cancer is an aggressive disease and the third highest cause of cancer-related mortality, with nearly 1,000,000 new cases occurring worldwide each year (1). Effective early diagnosis has led to prolonged survival. However, gastric cancer is typically diagnosed as advanced disease (2). Despite improving surgical and adjuvant therapies, the prognosis of patients with advanced gastric cancer remains dismal (3). The poor prognosis of patients with advanced gastric cancer is predominantly the result of the high rate of tumor metastasis and recurrence after curative resection (4, 5). Gastric cancer metastasis is a complex and multistep process involving multiple factors and genes (6, 7). However, our current understanding of the molecular mechanism underlying gastric cancer metastasis is far from complete. Much hope is focused on increasing our understanding of the signaling pathways and underlying biology involved in gastric cancer metastasis in order to develop new therapeutic options. Therefore, it is crucial to identify novel genes that govern gastric cancer metastasis and present predictive value for prognosis.

To identify novel candidates involved in gastric cancer metastasis, we used microarray based expression profiling of primary gastric cancer tissue samples with LNM (lymph node metastasis) and the samples without LNM. Using this high-throughput approach, we identified TIPRL (TOR signaling pathway regulator) as a novel candidate that was down-regulated in metastatic gastric cancer tissues through differential expression analysis.

TIPRL is an evolutionarily conserved protein which is identified as a homolog of yeast Tip41 (8). Unlike yeast TIP41, it has been shown that human TIPRL directly interacts with PP2A (Protein phosphatase 2A) and the PP2A-family phosphatases PP4 and PP6 (9, 10). It plays a key role in the ATM/ATR signaling pathway controlling DNA damage response and TOR (target of rapamycin) signaling through the regulation of PP2A (8, 9, 11). Recently, it has been reported that TIPRL is overexpressed in hepatocellular carcinoma, and that knockdown of TIPRL by small interfering RNA causes sustained activation of MKK7 (mitogen-activated protein kinase kinase 7) and JNK (c-Jun N-terminal kinase) by increasing MKK7 phosphorylation (12). This action of TIPRL appears to protect cancer cells from TRAIL (tumor necrosis factor-related apoptosis-inducing ligand)—induced apoptosis. However, detailed and mechanistic studies of the potential role of TIPRL in cancer invasion and metastasis are not available. Moreover, to date, no existing analyses have clarified the clinical and prognostic significance of TIPRL in human cancer, especially in gastric cancer. Therefore, in the current study, we investigated the gene expression, biological function, molecular mechanism and clinical significance of TIPRL in gastric cancer.

## Materials and Methods

### Patients and Tissue Specimens

After obtaining informed consent, 190 cases of paraffin-embedded tissues and 40 cases of fresh gastric cancer tissues, along with the available clinicopathological and follow-up information, were collected from patients who underwent curative resection of gastric cancer at Qilu Hospital of Shandong University from 2007 to 2014. All fresh samples were dissected from surgically resected specimens by pathologists at Qilu Hospital of Shandong University, and immediately snap-frozen in liquid nitrogen for the subsequent experiments. Histopathological diagnosis of each gastric cancer tissue was performed by the Department of Pathology, Qilu Hospital of Shandong University, according to the World Health Organization (WHO) criteria. Clinicopathological staging was classified on the basis of AJCC classification. None of the patients with gastric cancer had received adjuvant treatment before curative resection.

Our study was ethically-approved by the Ethics Committee of Shandong University, China. All subjects had provided informed consent.

### Gene Expression Microarray Analysis

Ten gastric cancer tissue samples (including 5 samples with LNM and 5 samples without LNM) with written informed consent were obtained. Total RNA from each gastric cancer tissue sample was isolated using TRIzol reagent (Invitrogen, Carlsbad, CA). After confirmation of RNA integrity and quantity, the RNA samples were analyzed at Kangchen Biotech (Kangchen, Shanghai, China) using Human LncRNA Array V2.0 (Arraystar, 8 × 60 K, Rockville, MD, USA) in accordance with the manufacturer's labeling, hybridization, scanning and normalization protocols. The criteria of significantly differentially expressed genes between the samples with LNM and the samples without LNM were a minimum of 2-fold absolute changes and a *P* < 0.05. More detailed information of the microarray data is available online via the NCBI's Gene Expression Omnibus (GEO) public database under the accession number GSE72307.

The mRNA expression profiles of MKN-45 cells treated with TIPRL overexpression plasmid or control were performed using the Human genome U133 Plus 2.0 Array (CapitalBio Corporation, Beijing, China). A fold change cutoff of 2.0 was set to identify differentially expressed mRNAs with biological significance between TIPRL-expressing MKN45 cells and empty vector controls.

### RNA Isolation and Real-Time Quantitative PCR

Total RNA from gastric cancer tissue samples or treated cells in log-phase was separately prepared using TRIzol reagent (Invitrogen), and the quality of RNA was estimated by NanoDrop Spectrophotometric analysis (NanoDrop Technologies, USA). A final amount of one microgram of total RNA for each sample was reversed-transcribed into first-strand complementary DNA (cDNA) using Transcriptor First-Strand cDNA Synthesis Kit (Roche). Real-time quantitative PCR was conducted in triplicate on cDNA templates using SYBR Green master mixture (Roche, Germany) in a volume of 10 μL on HT7900 system (Applied Biosystems, USA), with GAPDH as endogenous control. Relative quantification of target mRNA expression was evaluated by using the following equation 2^−ΔΔCt^. Primer sequences used in this assay are summarized in Table S1.

### Immunohistochemistry

For immunohistochemistry *in situ*, paraffin-embedded gastric cancer specimens (4-μm-thick) were sequentially cut, deparaffinized, and rehydrated. The standard SP (streptavidin-peroxidase-biotin) method (SP-9000 kit, ZSGB-bio, Beijing, China) was employed for immunohistochemical staining with a heat-induced epitope retrieval step, and endogenous peroxidases were blocked. Subsequently, tissue samples were incubated with rabbit polyclonal anti-TIPRL (1:250, ab70795, Abcam) at 4°C overnight, followed by detection with appropriate secondary antibodies. After washing, sections were visualized using DAB chromogen and counterstaining was carried out with hematoxylin. Images of immunostained sections were photographed and scored under a light microscope (Olympus, Japan).

TIPRL expression was evaluated in a semiquantitative method. For each specimen, immunostaining score of TIPRL was measured using a histochemical score (H-score), which takes extent and intensity of TIPRL staining in consideration. The extent score was determined on the basis of the percentage of positive tumor cells (0–100). The intensity score was graded from 0 to 3 (0, negative; 1, weak; 2, moderate; 3, strong). The extent and intensity scores were multiplied to obtain the final H-score (range 0–300), which represented the expression level of TIPRL. ROC (receiver operating characteristic) curve analysis was carried out to select the optimal cut-off value for TIPRL on the basis of the highest Youden's index (sensitivity + specificity −1).

### Cell Culture and Treatment

Two gastric cancer-derived cell lines, MKN45, and BGC823, were acquired from either the Shanghai Cancer Institute (Shanghai, China) or American Type Culture Collection (Manassas, VA, USA) and authenticated by DNA profiling. The gastric cancer-derived cell lines were maintained in RPMI-1640 medium (HyClone, Logan, UT, USA) with 10% fetal bovine serum (Gibco) under standard culture conditions, according to the recommended culture method.

For TIPRL overexpression, the human TIPRL (GenBank accession number NM_152902.5) coding sequence lacking the 3′UTR was constructed and subcloned into the mammalian expression vector [pcDNA3.1 (+) (pcDNA3.1 (+)-TIPRL] by Biosune Biotech (Shanghai, China). The TIPRL plasmid was transfected into cells using Turbofect transfection reagent (Thermo) following the manufacturer's protocols, whereas the empty plasmid [pcDNA3.1 (+)] was used as negative controls.

For TIPRL knockdown, small interfering RNA (siRNA) targeting human TIPRL (TIPRL siRNA, si-TIPRL, Targeting CTACAACAGATCATATAGA) and non-specific scrambled small interfering RNA were synthesized from Ribobio (Guangzhou, China). The siRNAs against human TIPRL were transfected into the gastric cancer cells at 50 nmol with X-tremeGENE transfection reagent (Roche, USA) according to the manufacturer's recommendations.

After transient transfection, the gastric cancer cells were incubated for 48 h before the subsequent functional assays were performed. Overexpression or knockdown of TIPRL was confirmed by western blot analysis.

### Cell Viability and Proliferation Assay

The effect of TIPRL on cell viability was assessed by the MTS assay (Promega, USA). Treated and control gastric cancer cells were trypsinized, counted, and then plated in 96-well plates (~4 × 10^3^ transfected cells/well) in quintuplicate, in a final volume of 100 μL of complete medium. Cell viability was determined on days 1, 2, and 3 by examining the number of cells with MTS labeling reagent.

Cell proliferation was detected by the 5′Ethynyl-2′-deoxyuridine (EdU) incorporation assay (Ribobio, Guangzhou, China). Briefly, treated and control cells in log-phase were trypsinized and seeded onto 96-well plates in triplicate at a density of 1 × 10^4^ cells/well the day before EdU incubation. After 12–24 h, EdU labeling solution (50 μmol) was added, and then treated and control cells were incubated for additional 2 h. After EdU incubating, cells were dyed with Apollo reaction cocktail and subsequently stained with 4′, 6-diamidino-2-phenylindole (DAPI). EdU positive cells were photographed and calculated with a fluorescence microscope (Olympus, Japan).

### Apoptosis Assay

Apoptosis was assessed by flow cytometry, using an Annexin V-FITC/PI double stain Kit (BestBio, Shanghai, China) according to the standard protocols. Floating and trypsinized adherent cells were harvested at 48-h post-transfection. After washing with chilled PBS, unfixed tumor cells were resuspended in binding buffer and stained with Annexin-V-FITC and PI. The stained samples were immediately detected with a FACScan flow cytometer (Beckman-Coulter, Los Angeles, CA, USA) for early and late apoptosis analysis.

### Cell Migration and Invasion Assays

The migratory and invasive potential of gastric cancer cells was assessed by using 24-well modified Boyden chambers (Corning, USA) with the polyethylene terephthalate membranes either uncoated or precoated with diluted Matrigel matrix (BD Biosciences, USA). After the appropriate treatments, MKN45 or BGC823 cell suspensions (1 × 10^5^ cells/well) in 200 μL of serum-free medium were transferred and cultured in each upper insert. Meanwhile, medium containing 10% FBS (500 μL) was applied to the lower compartment to induce migration or invasion in 24-well plates. After 24 h, Non-migrating or non-invading cells on the upper chambers were removed, whereas cells that had migrated or invaded through the lower side of the inserts were fixed in paraformaldehyde, rinsed with distilled water, stained with crystal violet, counted, and photographed under an inverted microscope (Nikon, Japan).

### Luciferase Assay

A fragment of human TIPRL-3′-UTR and the same fragment of TIPRL-3′-UTR with the miR-216a-5p/miR-383-5p putative binding site completely mutated was constructed by Biosune Biotech (Shanghai, China) and separately inserted into a pmirGLO vector (Promega), to synthesize a series of wild-type TIPRL-3′-UTR vectors (WT 3′-UTR) and mutant-type TIPRL-3′-UTR vectors (MUT 3′-UTR). Cells were co-transfected with a mix containing miRNA mimics (20 nmol) or negative control (GenePharma Biotech, China) and wild-type TIPRL-3′-UTR vector (20 ng) or mutant-type TIPRL-3′-UTR vector using Turbofect transfection reagent (Thermo). Forty-eight hours after transfection, cell lysates were prepared, and then renilla and firefly luciferase signals were calculated using the Dual-Luciferase Reporter system (Promega).

### PP2A Phosphatase Activity Assay

The MKN45 and BGC823 cells were transfected with TIPRL-expressing vector (pcDNA3.1 (±)-TIPRL), TIPRL siRNA, and the respective control vector. Samples from cells were prepared at 48-h post-transfection. The effect of TIPRL on PP2A activity was assessed by the phosphatase activity assay, using a PP2A Colorimetric Assay Kit (GENMED, Shanghai, China) in accordance with the manufacturer's protocols. Absorbance of each sample was measured at 660 nm using a microplate reader.

### Western Blot Analysis

In brief, the pellets of treated cells were dissolved in prechilled RIPA cell lysis buffer (BestBio, Shanghai, China), supplemented with phosphatase-inhibitor (Roche) and protease-inhibitor (BestBio). The lysate was purified by centrifugation and then cell debris was removed. The supernatant was collected until analysis and protein concentration was quantified by using the Bradford assay (Beyotime Biotechnology, China). Total protein extracts (30 μg) was fractionated by electrophoresis in denaturing 10 or 14% SDS-PAGE and transferred to PVDF transfer membranes (Millipore). Non-specific binding sites were blocked and blots were incubated with commercially available antibodies overnight at 4°C. Commercial primary antibodies used were as follows: rabbit polyclonal anti-TIPRL (1:4,000, ab70795, Abcam), rabbit monoclonal anti-phospho-AMPK (1:5,000, ab133448, Abcam), rabbit monoclonal anti-AMPK (1:1,000, ab207442, Abcam), rabbit monoclonal anti-phospho-mTOR (1:5,000, ab109268, Abcam), rabbit monoclonal anti-mTOR (1:1,000, #2983, Cell Signaling), rabbit monoclonal anti-phospho-p70 S6 Kinase (1:1,000, #9234, Cell Signaling), rabbit polyclonal anti-p70 S6 Kinase (1:2000, 14485-1-AP, proteintech), rabbit monoclonal anti-phospho-4E-BP1 (1:1000, #2855, Cell Signaling), rabbit monoclonal anti-4E-BP1 (1:5,000, ab32024, Abcam), and rabbit polyclonal anti-β-actin (1:10,000, AP0060, Bioworld). Bound antibodies were detected and visualized using the chemiluminescent substrate (Millipore, USA).

### Statistical Analysis

Results are analyzed as means ± SD from 3 representative independent experiments. Comparisons of continuous variables between two groups were carried out using Student's *t*-test. The correlation between the clinicopathologic categorical variables of patients with gastric cancer and TIPRL intensity scores was examined with the Chi-square test. Overall survival (OS) or disease-free survival (DFS) in relation to TIPRL expression was estimated by Kaplan-Meier method. Significance of differences between the low and high TIPRL expression groups was subsequently determined by applying log-rank test. Cox proportional hazards model was conducted to identify independent factors of survival. Statistical analysis and data plotting were conducted by using SPSS version 23.0 or GraphPad Prism 5. *P* < 0.05 was considered significant.

## Results

### Genome-Wide Microarray Analysis Identified TIPRL as a Metastasis-Related Gene in Human Gastric Cancer

**Figure 1:**
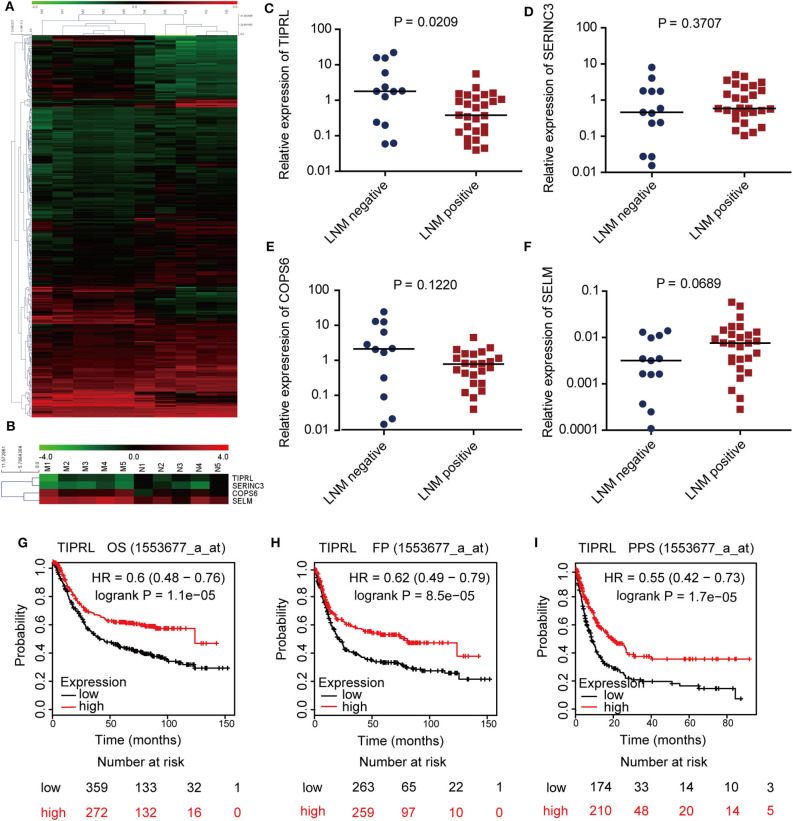
Identification and expression evaluation of TIPRL as a metastasis-related gene in gastric cancer. **(A,B)** Microarray plots of metastatic tumors (M group) vs. non-metastatic tumors (*N* group) demonstrating a different expression profile. Hierarchical cluster analysis shows 372 genes are significantly altered in gastric cancer samples with or without lymphatic metastasis (**A**, *P* < 0.05 with >2-fold change, raw value >500). Four novel genes (TIPRL, SERINC3, COPS6, and SELM) are significantly differentially expressed in metastatic gastric cancer tissues compared with their counterparts **(B)**. Each column represents a sample; each row denotes the expression level of a single gene. Expression level is demonstrated by colors: Green, underexpressed genes; red, overexpressed genes. **(C–F)** The mRNA expression levels of TIPRL, but not SERINC3, COPS6, and SELM, were significantly lower in gastric cancer tissues with LNM (positive) than those without LNM (negative), as determined by RT-qPCR (*t*-test, *P* = 0.0209, *P* = 0.3707, *P* = 0.1220, and *P* = 0.0689, respectively). **(G–I)** Low mRNA expression of TIPRL was correlated with poor overall survival (**G**, *P* = 1.1e-5), first progression survival (**H**, *P* = 8.5e-5), and post-progression survival (**I**, *P* = 1.7e-5) in gastric cancer patients from Kaplan-Meier plotter (http://kmplot.com/).

To identify novel gastric cancer-related candidates, genome-wide microarray was performed to compare the differential gene expression profiles of metastatic and non-metastatic cancer tissues of gastric cancer patients. Using stringent criteria, hundreds of differentially expressed genes were identified between metastatic and non-metastatic gastric cancer tissues (Figure 1A, *P* < 0.05 with >2-fold change, raw value >500; raw data accessible via GEO number: GSE72307). Based on the mining of microarray data, we focused on the novel genes, which have been poorly investigated and remained functionally uncharacterized in human cancer, especially in gastric cancer, because novel genes usually provide new insight into understanding human cancer. For this reason, TIPRL, SERINC3, COPS6, and SELM were chosen for further study (Figure 1B).

To further explore the microarray data, we evaluated the transcriptional levels of TIPRL, SERINC3, COPS6, and SELM in 40 frozen gastric tissues of gastric cancer patients by real-time PCR (Figures 1C–F). The statistical analysis revealed that TIPRL expression was significantly suppressed in metastatic compared with non-metastatic tissues, consistent with our microarray database (Figure 1C, *P* = 0.0209). Furthermore, data mining of the prognostic effect of TIPRL mRNA expression from Kaplan-Meier plotter (http://kmplot.com/) confirmed that lower TIPRL expression was associated with poor overall survival (OS), first progression survival (FP), and post-progression survival (PPS) in gastric cancer patients (Figure 1G, *P* = 1.1e-5, overall survival; Figure 1H, *P* = 8.5e-5, first progression survival; Figure 1I, *P* = 1.7e-5, post-progression survival). The data implied that an aberrant down-regulation of TIPRL might give rise to gastric cancer metastasis. Therefore, further investigations of TIPRL were instigated.

### TIPRL Was Significantly Down-Regulated and Associated With Gastric Cancer Clinicopathologic Features

**Figure 2:**
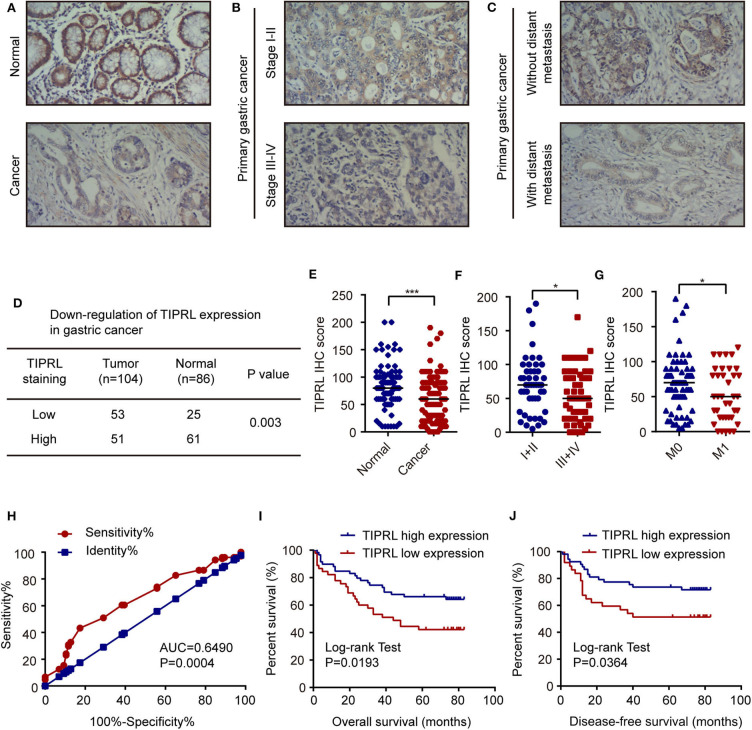
Decrease of TIPRL expression correlates with a poor clinical outcome in gastric cancer. **(A–C)** Representative immunohistochemical staining for TIPRL expression in normal gastric tissues, gastric cancer with different TNM tumor stages (I-II vs. III-IV) and gastric tumors with or without distant metastasis (M0 vs. M1). Original magnification, × 100. **(D)** Statistical analysis of TIPRL expression in gastric cancer tissues. **(E)** Quantitative analysis of TIPRL staining indicated that staining intensity in gastric tumors was significantly lower than normal gastric mucosa (*t*-test, ***P < 0.001). **(F)** TIPRL expression was dramatically decreased in patients at advanced stages (III-IV), in contrast with those at earlier stages (I-II) (*t*-test, *P < 0.05). **(G)** Analysis of TIPRL staining intensity also showed lower staining intensity in patient samples with distant metastasis (M1) compared to those without distant metastasis (M0) (*t*-test, *P < 0.05). **(H)** The ROC curves demonstrated strong separation between normal and gastric cancer tissues [AUC = 0.6490, CI (95%): 0.5709–0.7270, *P* = 0.0004]. The sensitivity and specificity of TIPRL expression to distinguish normal from gastric cancer tissues were 43.27 and 82.56%, respectively. **(I,J)** Kaplan–Meier survival curves revealed that low intensity of TIPRL immunostaining strongly correlated with poor overall survival (**I**, log-rank test, *P* = 0.0193) and disease-free survival (**J**, log-rank test, *P* = 0.0364).

Expression of TIPRL was also investigated by immunohistochemistry (IHC) in 104 gastric cancer samples and 86 paired non-tumor samples. IHC assays showed that TIPRL was predominantly localized in the cytoplasm (Figures 2A–C). Similarly, assessment via IHC revealed that TIPRL protein expression was markedly down-regulated in gastric tumors compared with their normal counterparts (Figures 2A,D,E). Moreover, intensity of TIPRL staining was significantly decreased in the advanced stage (III+IV) group, compared to the early stage (I+II) group (Figures 2B,F, *P* = 0.0467, Table 1). More importantly, semiquantitative analysis also showed lower levels of TIPRL expression in the tumors with distant metastasis, compared with those without distant metastasis (Figures 2C,G, *P* = 0.0083, Table 1).

**Table 1:** Correlation between TIPRL expression and clinicopathological features.

**Variables**	***n***	**TIPRL expression**		***P*-value**
		**Low**	**High**	
**Age (years)**				
≤62	56	20	36	0.1139
>62	48	25	23	
**Gender**				
Male	24	11	13	0.8171
Female	80	34	46	
**Tumor size (cm)**				
≤5	55	22	33	0.5535
>5	49	23	26	
**Clinical Stage**				
I/II	47	15	32	0.0467^*^
III/IV	57	30	27	
**Depth of Invasion (T)**				
T1	4	0	4	0.1778
T2	60	27	33	
T3	35	14	21	
T4	4	3	1	
Missing	1	1	0	
**Lymph Node Metastasis (LNM)**				
Negative (N0)	34	13	21	0.6699
Positive (N1–N3)	64	28	36	
Missing	6	4	2	
**Distant Metastasis (M)**				
Negative (M0)	66	22	44	0.0083^**^
Positive (M1)	38	23	15	
**Differentiation**				
Well	1	0	1	0.1542
Moderate	38	13	25	
Poor	62	31	31	
Missing	3	1	2	
**Prognosis**				
Survival	57	19	38	0.0297^*^
Death	47	26	21	

* P < 0.05 and

** *P < 0.01*.

Moreover, in receiver operator characteristic (ROC) curve analysis, which plots the area under the curve (AUC) to evaluate the diagnostic value of TIPRL in gastric cancer, clear separation was observed between normal and gastric cancer tissues, with an area of 0.6490 [Figure 2H, CI (95%): 0.5709–0.7270, *P* = 0.0004].

The correlation between protein expression of TIPRL and clinicopathologic features was further investigated using informative IHC data. The low and high levels of TIPRL expression in tissue samples were determined by ROC analysis, which demonstrated the optimal cut-off point of TIPRL is 55 (Figure 2H). After dichotomization based on the optimal cut-off value of TIPRL, low expression of TIPRL was positively correlated with advanced stages (*P* = 0.0467; Table 1), distant metastasis (*P* = 0.0083; Table 1), and poor prognosis (*P* = 0.0297; Table 1). However, TIPRL expression was not associated with age (*P* = 0.1139; Table 1), gender (*P* = 0.8171; Table 1), tumor size (*P* = 0.5535; Table 1), depth of invasion (*P* = 0.1778; Table 1), lymph node metastasis (*P* = 0.6699; Table 1), or tumor histological differentiation (*P* = 0.1542; Table 1). These data suggested that down-regulation of TIPRL might contribute to gastric cancer metastasis and progression.

### Low TIPRL Expression Predicted Poor Prognosis in Gastric Cancer Patients

Kaplan-Meier analysis was conducted to assess the correlation of TIPRL expression with gastric cancer prognosis. The survival analysis revealed that low expression of TIPRL was significantly correlated with shorter overall survival (OS) and disease-free survival (DFS) (Figures 2I,J, *P* = 0.0193 and *P* = 0.0364, respectively, log-rank test). In univariate Cox regression analysis, expression of TIPRL was correlated with OS and DFS in gastric cancer after curative resection [OS: HR = 0.512, CI (95%): 0.288–0.910, *P* = 0.023; DFS: HR = 0.473, CI (95%): 0.240–0.993, *P* = 0.031, Table 2]. Apart from TIPRL expression, tumor TNM stage (*P* = 0.000 and *P* = 0.000, respectively), depth of invasion (*P* = 0.001 and *P* = 0.006, respectively), lymph node metastasis (*P* = 0.000 and *P* = 0.002, respectively), distant metastasis (*P* = 0.000 and *P* = 0.000, respectively), and tumor histological differentiation (*P* = 0.017 and *P* = 0.004, respectively) were also significant predictors of outcome. In addition, multivariate analysis also revealed that distant metastasis, as well as tumor histological differentiation, were independent predictors of OS (*P* = 0.036) and DFS (*P* = 0.012) in gastric cancer, respectively. In all, our findings strongly suggest that loss of TIPRL is associated with invasion, metastasis, and an increased risk of poor prognosis in gastric cancer.

**Table 2:** Univariate and multivariate analysis of OS and DFS in gastric cancer patients.

**Variables**	**Univariate analysis**			**Multivariate analysis**		
	**HR**	**CI (95%)**	***P-*value**	**HR**	**CI (95%)**	***P-*value**
**Overall Survival**						
TIPRL expression	0.512	0.288–0.910	0.023^*^	0.956	0.500–1.827	0.892
Clinical stage	4.693	2.890–7.619	0.000	2.207	0.842–5.788	0.108
Depth of invasion	2.120	1.377–3.263	0.001	0.831	0.468–1.476	0.528
Lymph node metastasis	5.320	2.083–13.586	0.000	1.675	0.584–4.808	0.337
Distant metastasis	20.918	9.495–46.082	0.000	5.957	1.125–31.543	0.036
Differentiation	2.165	1.147–4.084	0.017	2.13	0.962–4.719	0.062
**Disease-Free Survival**						
TIPRL expression	0.473	0.240–0.993	0.031^*^	1.036	0.489–2.195	0.926
Clinical stage	6.182	3.223–11.858	0.000	2.747	0.736–10.253	0.133
Depth of invasion	2.017	1.223–3.326	0.006	0.736	0.401–1.451	0.409
Lymph node metastasis	5.152	1.800–14.747	0.002	0.945	0.290–3.076	0.925
Distant metastasis	33.734	12.354–92.117	0.000	8.969	0.918–87.589	0.059
Differentiation	3.675	1.518–8.896	0.004	3.854	1.339–11.088	0.012

*HR, hazard ratio; CI, confidence interval*.

* *P < 0.05*.

### Regulation of TIPRL by miR-216a-5p and miR-383-5p

**Figure 3:**
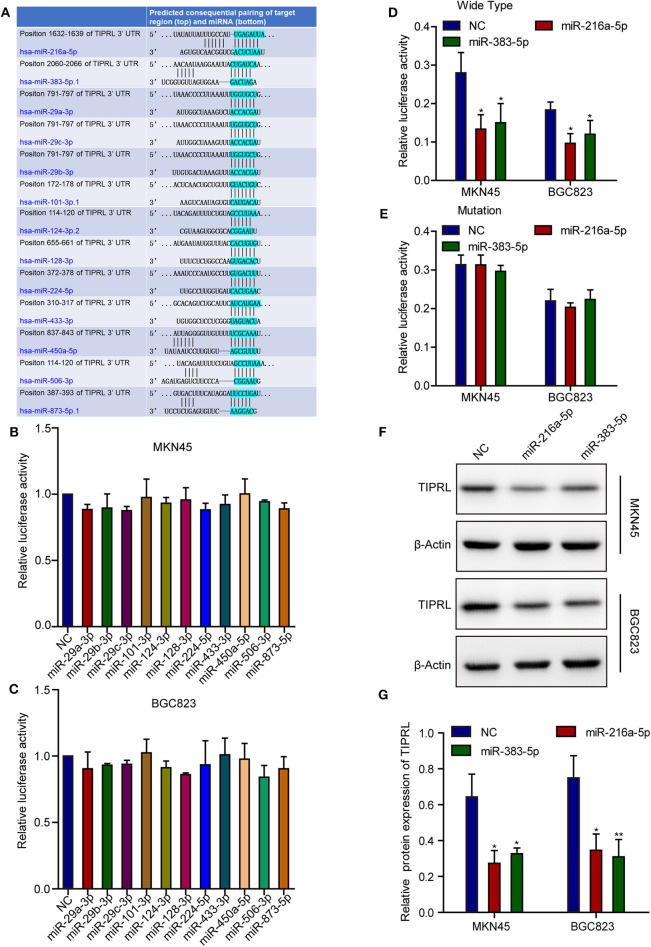
MiR-216a-5p and miR-383-5p repress TIPRL expression through directly targeting its 3′-UTR. **(A)** Schematic illustration of the putative miRNAs binding sites in 3′-UTR of TIPRL. **(B–E)** The wild-type and mutant form of TIPRL 3′-UTR regions were fused with a luciferase reporter (pmirGLO) and luciferase reporter assay was performed. MiR-216a-5p/383-5p rather than other miRNAs significantly inhibited the luciferase activity of wild-type TIPRL 3′-UTR reporters in MKN45 and BGC823 cells (**B–D**, *t*-test, *P < 0.05). Meanwhile, the luciferase responsiveness to miR-216a-5p/383-5p was abrogated by mutation of TIPRL 3′-UTR **(E)**. **(F,G)** Western blot analysis showed that up-regulated expression of miR-216a-5p/383-5p resulted in decreased protein expression of TIPRL. (*t*-test, *P < 0.05; **P < 0.01).

Post-transcriptional regulation involving miRNAs may contribute to TIPRL expression. Based on a literature review of the candidate miRNAs' function, TargetScan (http://www.targetscan.org/) and microRNA.org (http://www.microrna.org/) analysis revealed that the TIPRL 3′-untranslated region (3′-UTR) contained cancer-related miRNAs-binding sites, including miR-216a-5p, miR-383-5p, miR-29a-3p, miR-29b-3p, miR-29c-3p, miR-101-3p, miR-124-3p, miR-128-3p, miR-224-5p, miR-433-3p, miR-450a-5p, miR-506-3p, and miR-873-5p (Figure 3A). Next, Luciferase reporter assays were conducted to confirm the direct binding affinity between the candidate miRNAs and 3′-UTR of TIPRL. Both miR-216a-5p and miR-383-5p rather than the other 11 miRNAs dramatically impaired the luciferase activity of the wild-type reporter genes for TIPRL 3′-UTR in both MKN45 and BGC823 cells (Figures 3B–D), but there was no remarkable change in the relative luciferase activity in cells encompassing the mutant binding site of TIPRL (Figure 3E). Furthermore, Western blot analysis further confirmed that ectopic expression of either miR-216a-5p or miR-383-5p resulted in decreased protein expression of TIPRL in both MKN45 and BGC823 cells (Figures 3F,G). Our findings indicate that miR-216a-5p and miR-383-5p could directly recognize binding sites in TIPRL 3′-UTR, and TIPRL is down-regulated by miR-216a-5p and miR-383-5p in gastric cancer cells.

### TIPRL Impaired Migratory and Invasive Capacities of Gastric Cancer Cells *in vitro*

**Figure 4:**
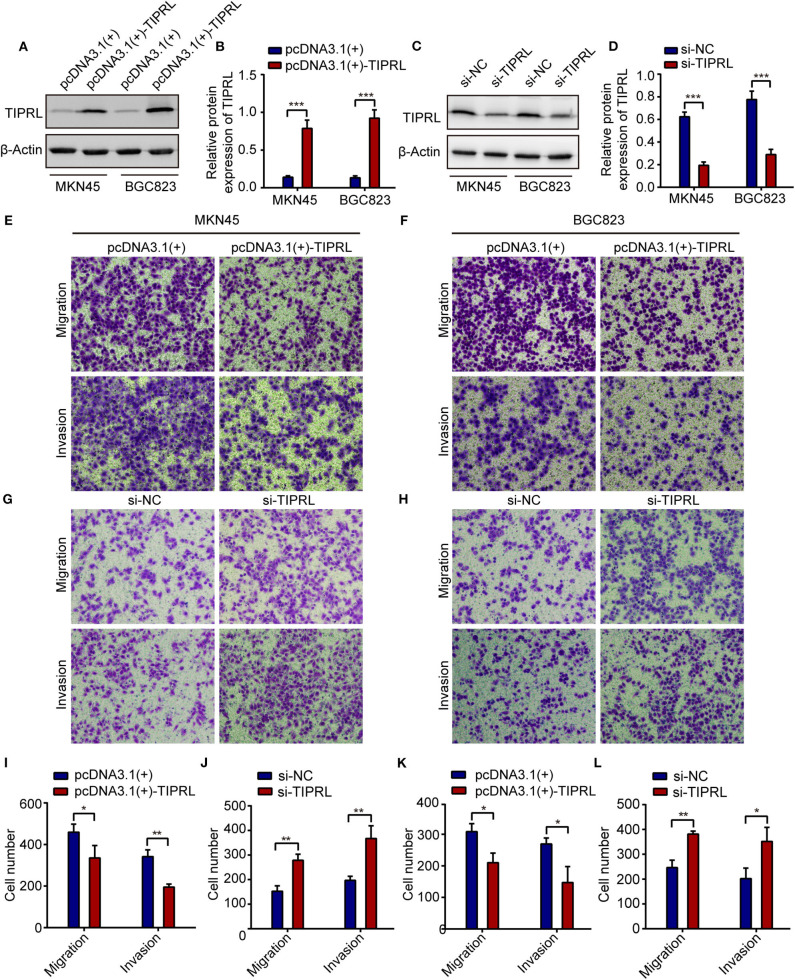
TIPRL attenuates migration and invasion of gastric cancer cells. **(A–D)** The MKN45 and BGC823 cells were transfected with TIPRL-expressing vector [pcDNA3.1 (+)-TIPRL], TIPRL siRNA, and the respective control vector. The protein levels of TIPRL were examined by WB (***P < 0.001). **(E,F,I,K)** Expression of TIPRL inhibited the migration and invasion of MKN45 and BGC823 cells, as determined by transwell migration and matrigel invasion assays (*t*-test, *P < 0.05; **P < 0.01). **(G,H,J,L)** siRNA-mediated knockdown of TIPRL promoted the migration and invasion of MKN45 and BGC823 cells (*t*-test, *P < 0.05; **P < 0.01). All experiments were performed in triplicate. Error bars, SD.

To substantiate the possible role of TIPRL in regulating gastric cancer tumorigenesis and progression, we adopted gain-of-function and loss-of-function assays to investigate TIPRL function in gastric cancer. First, TIPRL expression vector or empty vector was transiently transfected into MKN45 and BGC823 cells. Conversely, we genetically decreased the expression of TIPRL in MKN45 and BGC823 cells with TIPRL-specific siRNAs. The up-regulation and knockdown of TIPRL expression were evidenced by western blotting (Figures 4A–D). In addition, the effects of TIPRL on migration and invasion of gastric cancer cells were assessed by transwell assays. The ectopic expression of TIPRL markedly inhibited the migration capacities of MKN45 and BGC823 cells compared with respective empty vector-transfected MKN45 and BGC823 cells (Figures 4E,F,I,K). Matrigel invasion assay also revealed that the forced expression of TIPRL significantly reduced cell invasion in MKN45 and BGC823 cells (Figures 4E,F,I,K). Meanwhile, an inverse effect was observed in MKN45 and BGC823 cells with silencing TIPRL expression (Figures 4G,H,J,L). In concordance with the clinical and prognostic significance of TIPRL in gastric cancer patients, the above results illustrate that TIPRL is a critical regulator of migration and invasion in gastric cancer cells.

### Effect of TIPRL on Cell Proliferation and Apoptosis of Gastric Cancer Cells

**Figure 5:**
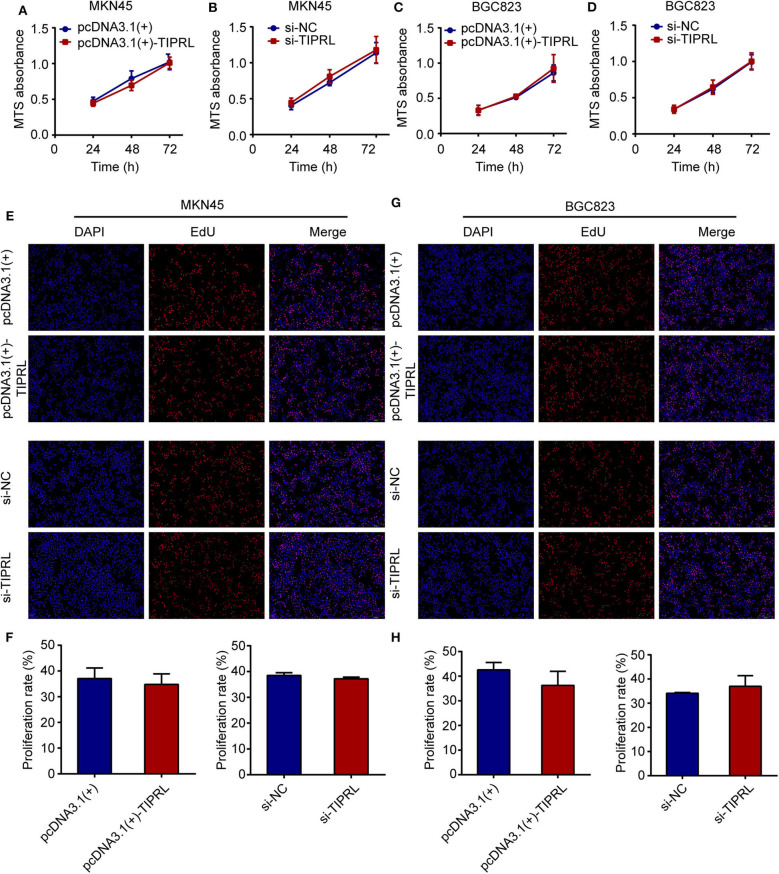
Effect of TIPRL on cell proliferation in gastric cancer cells. **(A–D)** After transfection, cell viability was determined by MTS assay. The data showed that TIPRL had no effect on cell viability of MKN45 and BGC823 cells (*t*-test, *P* > 0.05). **(E–H)** EDU assays demonstrated that TIPRL did not influence the proliferation activities in MKN45 and BGC 823 cells (*t*-test, *P* > 0.05). All experiments were performed in triplicate. Error bars, SD.

**Figure 6:**
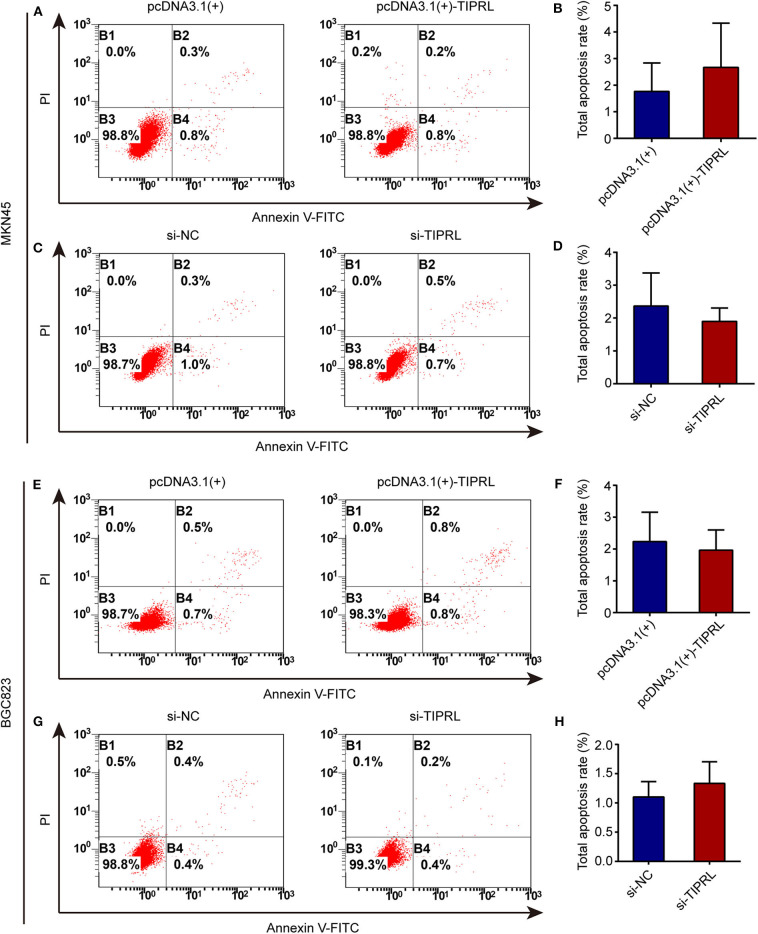
Effect of TIPRL on cell apoptosis in gastric cancer cells. **(A–H)** Up-regulation or down-regulation of TIPRL expression could not induce apoptosis in both cell lines, as indicated by flow cytometry analysis following Annexin V-FITC/PI staining (*t*-test, *P* > 0.05). All experiments were performed in triplicate. Error bars, SD.

To investigate the effect of TIPRL on gastric cancer cell proliferation and survival, MTS, EdU, and cell apoptosis assays were performed. The exogenous expression of TIPRL could not have a considerable effect on cell viability in MKN45 and BGC823 cells, while a similar effect was observed in MKN45 and BGC823 with silencing TIPRL expression (Figures 5A–D). In keeping with this, ectopic expression of TIPRL or knockdown of TIPRL did not affect cell proliferation, as evidenced by EdU proliferation assay in MKN45 and BGC823 (Figures 5E–H). Additionally, apoptosis analysis by flow cytometry revealed that proportions of apoptotic cells were similar between TIPRL overexpressed or TIPRL siRNA cells and respective controls in both cell lines (Figures 6A–H).

### TIPRL Suppressed Invasion and Migration Through Regulation of AMPK/mTOR Pathway

**Figure 7:**
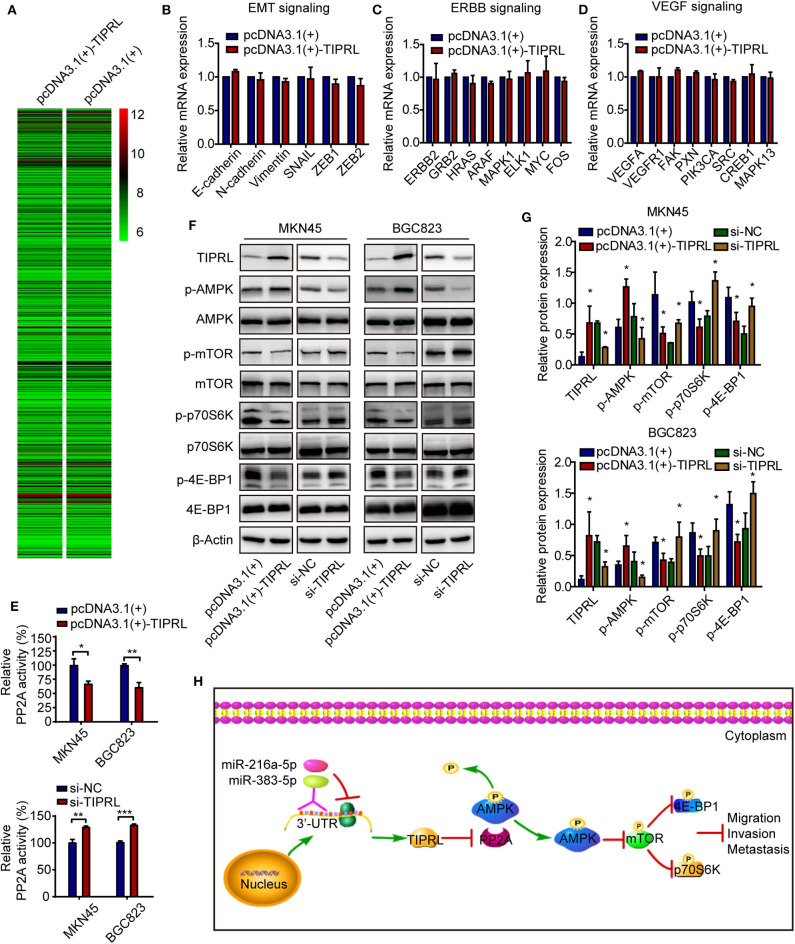
TIPRL suppresses metastatic potential of gastric cancer via AMPK/mTOR signaling. **(A)** Microarray study on TIPRL-regulated genes. Hierarchical clustering of TIPRL-regulated genes on the basis of the expression patterns. Almost no change was observed at the mRNA levels between TIPRL overexpression and control cells. **(B–D)** Validation of the microarray analysis. No significant change at the mRNA expression levels of genes related to EMT, ERBB, and VEGF signaling pathways in MKN45 cells transfected with TIPRL overexpression and control vector was further validated using RT-qPCR. **(E)** PP2A activity analysis indicated that TIPRL resulted in inhibition of PP2A activity by PP2A activity assay (*t*-test, *P < 0.05; **P < 0.01; and ***P < 0.001). Overexpression of TIPRL significantly reduced PP2A activity in MKN45 and BGC823 cells. Conversely, an inverse effect was observed in MKN45 and BGC823 cells with silencing TIPRL expression. **(F,G)** Immunoblot results showed that up-regulated expression of TIPRL enhanced phosphorylation of AMPK (p-AMPK) and reduced phosphorylation of mTOR (p-mTOR), p70S6K (p-p70S6K), and 4E-BP1 (p-4E-BP1); Down-regulated expression of TIPRL led to the opposite changes (*t*-test, *P < 0.05). The total protein levels of AMPK, mTOR, p70S6K, and 4E-BP1 did not change. **(H)** Schematic illustration of the molecular basis of TIPRL as a metastasis suppressor in gastric cancer. TIPRL, a target of miR-216a-5p/383-5p, facilitates AMPK phosphorylation via preventing the PP2A-dependent dephosphorylation and inactivation of AMPK, which in turn attenuates phosphorylation of mTOR and its downstream effectors p70S6K and 4E-BP1, leading to inactivation of mTOR signaling and subsequent suppression of invasion and metastasis of gastric cancer.

To explore the molecular mechanism underlying the anti-invasive function of TIPRL, gene expression in TIPRL and vector transfected MKN45 cells were analyzed using whole-genome mRNA microarray. Unexpectedly, compared with empty vector-transfected cells, only 4 down-regulated genes (IGFBP1, NDRG1, EIF4G2, and NBPF10; fold change ≥2), which were not metastasis-related gene in human cancer, were detected in the MKN45 cells overexpressing TIPRL, microarray analysis revealed that almost all the genes remained unaffected at the mRNA levels, suggesting that TIPRL may modulate the genes at the post-transcriptional levels (Figure 7A). In addition, no significant change at the mRNA levels was further validated using RT-qPCR by specific primers available from our laboratory (Figures 7B–D), supporting the reliability of the microarray analysis. Intriguingly, TIPRL has been identified as a pivotal inhibitory regulator of protein phosphatase 2A (PP2A) (9, 10), and PP2A contributes significantly to AMP-activated protein kinase (AMPK) inactivation by dephosphorylation (13). Based on the interaction between TIPRL and PP2A, we hypothesized that TIPRL might exert the inhibitory effect on cell migration/invasion through activating AMPK signaling by inhibition of PP2A. We further evaluated the effect of TIPRL on PP2A activity in MKN45 and BGC823 cells. Consistently, TIPRL overexpression significantly reduced PP2A activity, whereas TIPRL silencing dramatically increased PP2A activity (Figure 7E). As expected, Western blot analysis confirmed this hypothesis and indicated that in the TIPRL-transfected MKN45 and BGC823 cells, phosphorylation of AMPK was markedly increased, while silencing TIPRL induced the opposite effects (Figures 7F,G). Moreover, as AMPK/mTOR signaling affects tumor invasion and metastasis (14–16), the AMPK downstream effectors of mTOR, p70S6K, and 4E-BP1 were also examined. Accordingly, phosphorylation of mTOR, p70S6K, and 4E-BP1 was substantially decreased in TIPRL expressed cells. Meanwhile, siRNA-mediated knockdown of TIPRL led to the opposite changes (Figures 7F,G). Furthermore, the total protein levels of AMPK, mTOR, p70S6K, and 4E-BP1 were not significantly affected under either condition (Figure 7F). Thus, these results support our notion that TIPRL suppresses cell migration/invasion of gastric cancer through regulating AMPK/mTOR signaling pathway.

## Discussion

Invasion and metastasis of gastric cancer after curative resection remain the most common lethal outcomes with few efficacious therapeutic options. Therefore, it is critical to understand the mechanisms underlying gastric cancer metastasis in order to discover novel effective therapeutic targets for clinical evaluation.

Using microarray analysis of metastatic and non-metastatic tumors, we identified TIPRL as a novel metastasis suppressor in gastric cancer through gene expression microarray. TIPRL is a ubiquitously expressed protein which functions as a key inhibitory regulator of PP2A-like phosphatases, including PP2A, PP4, and PP6 (9, 10). Despite this, little is known about the functional and prognostic implications of TIPRL in cancer, particularly in tumor metastasis. Recently, a growing body of evidence supports an oncogenic role for PP2A-like enzymes. Upregulation of the catalytic subunit of PP2A predicts poor prognosis and promotes carcinogenesis through inhibition of p53 mediated apoptosis in hepatocellular cancer models (17, 18). In basal breast cancer, PP2A appears to act as a metastasis promoter by activating cofilin-1 (CFL-1) (19). In addition, PP4 has also been found to be overexpressed in numerous types of cancer (20–22) and inhibition of PP4 expression increases efficacy of cisplatin treatment (21). Given aforementioned studies and function of TIPRL, it is plausible to assume that TIPRL may be a potential tumor suppressor gene. Herein, we highlight a functional role for TIPRL in invasion and metastasis of gastric cancer.

The clinical relevance of TIPRL in gastric cancer was investigated in a large well-characterized clinical cohort. We showed that TIPRL was frequently decreased in gastric cancer tissues, relative to non-tumor tissues. Moreover, TIPRL expression was markedly down-regulated in primary tumor samples with distant metastasis, compared to those without distant metastasis. Importantly, IHC analysis of TIPRL in gastric cancer demonstrated a strong association between low expression of TIPRL and unfavorable clinicopathological variables such as more-advanced TNM stage and distant metastasis, suggesting that TIPRL down-regulation might facilitate a metastatic phenotype. Furthermore, significantly shortened overall survival and disease-free survival were observed in gastric cancer patients with low TIPRL expression compared with patients with high TIPRL expression. In keeping with our data, higher expression of TIPRL was associated with a favorable prognosis in gastric carcinoma patients according to analysis of publicly available data sets. These clinical data strongly suggested that TIPRL might be involved in the metastasis and progression of gastric cancer and serve as a novel useful prognostic biomarker.

Currently, regulatory mechanisms of TIPRL are not yet documented. MicroRNAs (miRNAs) play a pivotal role in tumorigenesis via negatively regulating target gene expression at the post-transcriptional level (23, 24). Using bioinformatics analysis and luciferase assay, we found that miR-216a-5p and miR-383-5p could directly regulate TIPRL expression through targeting the 3′-UTR of TIPRL. In recent years, accumulating evidence has demonstrated that miR-216a-5p and miR-383-5p are significantly elevated and function as oncogenic miRNAs in a variety of tumors (25–31). In particular, previous study have shown that miR-216a promotes invasion and metastasis in hepatocellular carcinoma through targeting TSLC1, PTEN, and SMAD7 (25–27), which is further confirmed in a variety of cell models (28–30). Moreover, it has been reported that miR-383 promotes cholangiocarcinoma cell invasion and proliferation by suppressing IRF1 (31). More importantly, in gastric cancer, miR-216a is significantly upregulated (32), and elevation of miR-216a would favor a worse clinical outcome (33). Additionally, elevated miR-216a-3p activates the NF-κB signaling pathway through targeting RUNX1, contributing to metastatic potential of gastric cancer (34). Here, our current study pointed out that miR-216a-5p/383-5p suppressed TIPRL expression, thus suggesting that elevation of miR-216a-5p/383-5p might contribute to aberrant down-regulation of TIPRL in gastric cancer. This study enriches our horizon of TIPRL regulation by miRNAs.

Our clinical data urged us to investigate the putative tumor-suppressive function of TIPRL in gastric cancer *in vitro*. Re-expression of TIPRL in MKN45 and BGC823 cells markedly suppressed the migration and invasion abilities; while the knockdown of TIPRL promoted migration and invasion of the gastric cancer cells *in vitro*. Both assays of forced and silenced expression of TIPRL revealed that TIPRL could suppress cell migration and invasion in gastric cancer, which are two crucial events during tumor metastasis (35), consistent with clinical observations. Moreover, previous report demonstrates that TIPRL prevents TRAIL-induced apoptosis through inactivation of MKK7-JNK signaling in hepatocellular carcinoma (12). However, in the current study, the apoptosis and proliferation of gastric cancer cells were not affected. Thus, the effects of TIPRL may be cell context-dependent. Collectively, these findings provide the first demonstration that TIPRL acts as a novel metastasis suppressor in gastric cancer.

We further elucidated the molecular basis by which TIPRL exerted the suppressive effect on cell migration and invasion in gastric cancer using mRNA microarray. Unexpectedly, it is noteworthy that TIPRL could not modulate the genes at transcriptional levels by the microarray and real-time PCR analysis, thereby implying that the regulation might occur at the post-transcriptional levels. Intriguingly, it is known that TIPRL has a well-established role as a crucial modulator to inactivate the phosphatase activity of PP2A (9, 10). PP2A, a major serine-threonine phosphatase, regulates a variety of kinase-driven intracellular signaling pathways by dephosphorylating many pivotal cellular molecules (36). The predominant form of PP2A inside cells contains a heterotrimer formed by catalytic (C), scaffolding (A), and regulatory (B) subunits (9). Structure-guided studies reveal that the butterfly-shaped TIPRL binds specifically to the PP2A catalytic subunit (C) and perturbs the phosphatase active site, resulting in phosphatase inactivation. TIPRL also makes dynamic wobble contacts with scaffolding (A) subunit, leading to enhanced inactivation of disease-associated mutant PP2A. More importantly, TIPRL and latency chaperone, alpha4, coordinate to promote disassembly of PP2A complexes (10). Consistently, our study indicated that TIPRL negatively regulated PP2A activity in gastric cancer cells. Moreover, PP2A, an upstream phosphatase of AMPK (13), directly interacts with AMPK and negatively regulates AMPK activity by dephosphorylating Thr-172, a residue that is required for AMPK activation when phosphorylated (37). AMPK activity is also negatively regulates by calcium-mediated PR72-containing PP2A (38). Additionally, previous reports demonstrate that subunit A of PP2A co-immunoprecipitates with AMPK (39), leading to inactivation of AMPK activity in a glucose-dependent manner (39, 40). Furthermore, targeting PP2A by LB-100 (a novel PP2A inhibitor) activates AMPK to suppress colorectal cancer in *vitro* and *in vivo* (41). Unsurprisingly, PP2A also negatively regulates AMPK signaling by dephosphorylating and inactivating AMPK. Given the interaction between TIPRL and PP2A, we therefore postulated that TIPRL might potentiate AMPK signaling via preventing the PP2A-dependent dephosphorylation and inactivation of AMPK. As a critical cellular energy sensor, AMPK plays a central role in regulating cellular metabolism and energy homeostasis (42). Augmented AMPK activity also contributes to suppression of invasive and metastatic capacities of cancer cells (16, 43), which is a key process during tumor progression. As expected, overexpression of TIPRL induced strong phosphorylation and activation of AMPK in gastric cancer cells, whereas an inverse effect was observed in TIPRL-deficient cells. Therefore, the suppression of cell migration and invasion induced by TIPRL in gastric cancer might attribute, at least in part, to the TIPRL-mediated phosphorylation/activation of AMPK signaling. Concomitantly, compelling evidence indicates that AMPK activation has emerged as a pivotal negative regulator of mTOR and its downstream effectors (14, 44, 45), which intimately relates to tumor invasion and metastasis (15, 46–50). Accordingly, it is of interest to determine the effect of TIPRL on mTOR and its downstream effectors p70S6K and 4E-BP1. These data indicated that TIPRL could attenuate phosphorylation of mTOR, p70S6K, and 4E-BP1, thereby suppressing the mTOR signaling pathway. Together, our findings suggest that TIPRL may induce phosphorylation/activation of AMPK, which in turn attenuates the mTOR pathway, leading to inactivation of mTOR signaling and subsequent suppression of cell migration/invasion in gastric cancer.

To date, the role of TIPRL in cancer has been documented only in liver and lung cancer (12, 51, 52). In hepatocellular carcinoma samples and cell lines, TIPRL is overexpressed and prevents TRAIL-induced apoptosis through inactivation of MKK7-JNK signaling (12), thereby representing a potential biomarker for early liver cancer (51). In addition, TIPRL overexpression is found to induce autophagy and accelerate growth through the eIF2α-ATF4 pathway in non-small cell lung cancer (52). Given the previous reports, TIPRL is believed to be oncogenic. However, our study indeed suggested that TIPRL functioned as a metastasis suppressor in gastric cancer through regulating AMPK/mTOR signaling. This indicates that TIPRL may have strikingly distinct functional roles in tumorigenesis depending on the cellular context. For example, the dual role of p21 as a tumor suppressor and an oncogene in different types of cancer has been documented (53). Thus, our findings may lead to further studies of the effects of TIPRL in other cancers.

Taken together, the present study provides the first evidence that TIPRL, a target of miR-216a-5p/383-5p, is identified as a potential metastasis suppressor gene in gastric cancer. Clinically, loss of TIPRL expression in gastric cancer is a strong indicator of metastatic phenotype and poor clinical outcomes. TIPRL exerts its anti-invasive function through regulating AMPK/mTOR signaling pathway (Figure 7H). Thus, TIPRL may represent a prognostic biomarker and a promising target for new therapies in gastric cancer.

## Data Availability Statement

The datasets presented in this study can be found in online repositories. The names of the repository/repositories and accession number(s) can be found in the article/Supplementary Material.

## Ethics Statement

The studies involving human participants were reviewed and approved by the Ethics Committee of Shandong University, China. The patients/participants provided their written informed consent to participate in this study.

## Author Contributions

PG designed and supervised the whole study and revised the manuscript. ML and S-SS performed the experiments and drafted the manuscript. D-BS, H-TL, and R-RM provided technical support and assisted with the experiments. X-QX and Y-JS conducted statistical analysis. All authors read and approved the final manuscript.

## Conflict of Interest

The authors declare that the research was conducted in the absence of any commercial or financial relationships that could be construed as a potential conflict of interest.
