# Supplementary material for: TIPRL, a Novel Tumor Suppressor, Suppresses Cell Migration, and Invasion Through Regulating AMPK/mTOR Signaling Pathway in Gastric Cancer
Source: Front Oncol. 2020 Jul 3;10:1062. doi: 10.3389/fonc.2020.01062 (PMC7350861; doi:10.3389/fonc.2020.01062)
Supplement: Supplementary file 1 [file Table_1.DOCX]

Supplementary Material

**Table S1. PCR primers used in this study.**

| **Gene** | **Sequence** |
| --- | --- |
| TIPRL-F | TGTTCCACCTTCCCTCTTCA |
| TIPRL-R | TTGTGAGTCTGCTGGGTTAGG |
| SERINC3-F | GCATCCGCACTTCCACTAAT |
| SERINC3-R | GGCTGTCCATCTTCTTCATCA |
| COPS6-F | CCACAATGCTGTTTGCTGA |
| COPS6-R | TCTCTCCACTGCCTGTTGC |
| SELM-F | CGAAGAGATCAATGCGCTAGT |
| SELM-R | GTGGTCCGAAGTTTCCTCTG |
| E-cadherin-F | CGAGAGCTACACGTTCACGG |
| E-cadherin-R | GGGTGTCGAGGGAAAAATAGG |
| N-cadherin-F | AGCCAACCTTAACTGAGGAGT |
| N-cadherin-R | GGCAAGTTGATTGGAGGGATG |
| Vimentin-F | GACGCCATCAACACCGAGTT |
| Vimentin-R | CTTTGTCGTTGGTTAGCTGGT |
| SNAIL-F | TCGGAAGCCTAACTACAGCGA |
| SNAIL-R | AGATGAGCATTGGCAGCGAG |
| ZEB1-F | CAGCTTGATACCTGTGAATGGG |
| ZEB1-R | TATCTGTGGTCGTGTGGGACT |
| ZEB2-F | CAAGAGGCGCAAACAAGCC |
| ZEB2-R | GGTTGGCAATACCGTCATCC |
| ERBB2-F | ACAACCAAGTGAGGCAGGTC |
| ERBB2-R | GGTATTGTTCAGCGGGTCTC |
| GRB2-F | GCCATCGCCAAATATGACTT |
| GRB2-R | TTCCATTAAGCTCTGCCTTGT |
| HRAS-F | GGAGTGGAGGATGCCTTCTA |
| HRAS-R | TCAGGAGAGCACACACTTGC |
| ARAF-F | AGCTGAGGTGATCCGTATGC |
| ARAF-R | TGTGGCTGTAAGGCAGTGAG |
| MAPK1-F | GTTGCAGATCCAGACCATGA |
| MAPK1-R | GACTTGGTGTAGCCCTTGGA |
| ELK1-F | CAAGCTGGTGGATGCAGAG |
| ELK1-R | TTGCGGATGATGTTCTTGTC |
| MYC-F | CCAGAGGAGGAACGAGCTAA |
| MYC-R | TTGGACGGACAGGATGTATG |
| FOS-F | GCTTCCCTTGATCTGACTGG |
| FOS-R | GCTGCTGATGCTCTTGACAG |
| VEGFA-F | GTCCAACTTCTGGGCTGTTCT |
| VEGFA-R | CTCACCCGTCCATGAGCCC |
| VEGFR1-F | CCAAATAAGCACACCACGCC |
| VEGFR1-R | TGCTTTGGTCAATTCGTCGC |
| FAK-F | GCTTACCTTGACCCCAACTTG |
| FAK-R | ACGTTCCATACCAGTACCCAG |
| PXN-F | CTGCTGGAACTGAACGCTGTA |
| PXN-R | GGGGCTGTTAGTCTCTGGGA |
| PIK3CA-F | CGTTTCTGCTTTGGGACAAC |
| PIK3CA-R | CCTGATGATGGTCGTGGAG |
| SRC-F | GAGCGGCTCCAGATTGTCAA |
| SRC-R | CTGGGGATGTAGCCTGTCTGT |
| CREB1-F | GCTGCCTCTGGAGACGTACAA |
| CREB1-R | GCTAGTGGGTGCTGTGCGA |
| MAPK13 | CTCACCCATCCCTTCTTTGA |
| MAPK13 | GCTGCTTCCATTCATCCACT |
